# The Inhibitory Effect of Pseudolaric Acid B on Gastric Cancer and Multidrug Resistance via Cox-2/PKC-α/P-gp Pathway

**DOI:** 10.1371/journal.pone.0107830

**Published:** 2014-09-24

**Authors:** Qian Sun, Yan Li

**Affiliations:** Department of gastroenterology, Shengjing Hospital Affiliated to China Medical University, Shenyang, Liaoning, China; Children's Hospital Boston & Harvard Medical School, United States of America

## Abstract

**Aim:**

To investigate the inhibitory effect of pseudolaric acid B on subcutaneous xenografts of human gastric adenocarcinoma and the underlying molecular mechanisms involved in its multidrug resistance.

**Methods:**

Human gastric adenocarcinoma SGC7901 cells and drug-resistant SGC7901/ADR cells were injected into nude mice to establish a subcutaneous xenograft model. The effects of pseudolaric acid B with or without adriamycin treatment were compared by determining the tumor size and weight. Cyclo-oxygenase-2, protein kinaseC-α and P-glycoprotein expression levels were determined by immunohistochemistry and western blot.

**Results:**

Pseudolaric acid B significantly suppressed the tumor growth induced by SGC7901 cells and SGC7901/ADR cells. The combination of pseudolaric acid B and the traditional chemotherapy drug adriamycin exhibited more potent inhibitory effects on the growth of gastric cancer in vivo than treatment with either pseudolaric acid B or adriamycin alone. Protein expression levels of cyclo-oxygenase-2, protein kinaseC-α and P-glycoprotein were inhibited by pseudolaric acid B alone or in combination with adriamycin in SGC7901/ADR cell xenografts.

**Conclusion:**

Pseudolaric acid B has a significant inhibitory effect and an additive inhibitory effect in combination with adriamycin on the growth of gastric cancer in vivo, which reverses the multidrug resistance of gastric neoplasm to chemotherapy drugs by downregulating the Cox-2/PKC-α/P-gp/mdr1 signaling pathway.

## Introduction

Gastric cancer is one of the world’s most common cancers [1], and chemotherapy is an important strategy against gastric cancer [2]. Because multiple chemotherapeutic drugs of different classes are used to treat gastric cancer, the sensitivity of anticancer drugs to gastric cancer decreases gradually, and resistance to many different drugs with different chemical structures and different mechanisms of action can occur. This type of resistance is called multidrug resistance (MDR) and significantly decreases the efficiency of therapy on gastric cancer. MDR is becoming a major problem for successful cancer treatment [3]. It has been discovered that the major reason for MDR in cancer is the overexpression of P-glycoprotein (P-gp), a product of the human MDR1 gene. P-gp is an ATP-dependent efflux pump that can decrease drug accumulation in cancer cells. It is proposed that P-gp, a well-accepted biomarker of MDR, should also be considered as a molecular target in multidrug-resistant cancer [4]. Recent studies have demonstrated that in cancer cells with MDR, the expression levels of cyclo-oxygenase-2 (Cox-2) and an isoform of protein kinaseC (PKC-α) are both upregulated, and their inhibition can reverse neoplastic MDR [5]–[10].

**Figure 1:**
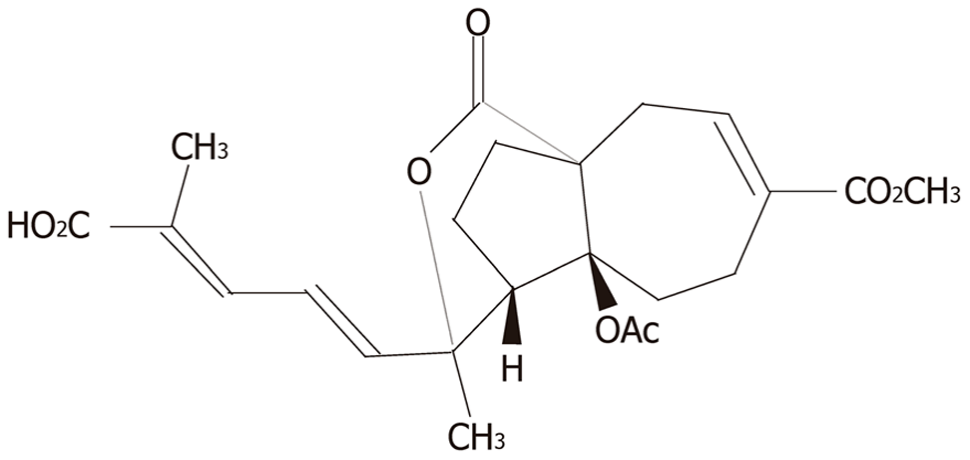
Chemical structure of pseudolaric acid B (C_23_H_28_O_8_, MW = 432.5).

Pseudolaric acid B (PAB, Figure 1), a diterpene acid isolated from the root and trunk bark of the tree *Pseudolarix kaempferi* Gordon (Pinaceae), is used in traditional Chinese medicine for its wide spectrum of biological features, such as anti-fungal effects and anti-fertility effects. In addition, its anti-angiogenic effects have also been gradually recognized in recent years [11], [12]. Recent studies have demonstrated that PAB induces growth inhibition, cell cycle arrest and apoptosis. Moreover, PAB reverses the multidrug resistance of cancer cells [13], [14]. PAB suppresses the growth of the gastric neoplastic AGS cell line and induces its apoptosis [15]. Our previous research demonstrated that PAB also has a dramatic inhibitory effect on human gastric cancer SGC7901 cells in vitro [16]. However, in vitro studies of the anticancer effect of PAB are rare, and neither the in vivo efficacy of this novel herbal compound against tumors nor its precise molecular mechanism against MDR has been fully investigated.

The aim of the present study is to evaluate the anti-neoplastic effect of PAB in vivo, including the reversal of MDR, using a xenograft model in nude mice and explore whether PAB’s underlying molecular mechanism is related to the inhibition of MDR via the Cox-2/PKC-α/P-gp pathway.

## Materials and Methods

### Materials

RPMI-1640 culture medium and fetal bovine serum (FBS) for cell culture were purchased from Gibco BRL, Gaithersburg, MD, USA. Rabbit anti-Cox-2 monoclonal antibody, mouse anti-PKC-α and mouse anti-P-gp monoclonal antibodies were purchased from Santa Cruz Corporation, CA, USA. Secondary antibodies against the rabbit and mouse antibodies, streptavidin-peroxidase (SP) kits, and 3,3’-diaminobenzidine (DAB) were obtained from Beijing Zhongshan Biotechnology, China. BCA protein assay kits and SDS-PAGE gel preparation kits were obtained from the Beyotime Institute of Biotechnology, Shanghai, China. PAB was purchased from the Liaoning Institute for Drug Control, China No. 201003 (the purity:>98%). Adriamycin (ADR) was obtained from Zhejiang Hisun Pharmaceutical Co., Ltd., China No. 120906. Tween80 and polyethylene glycol were purchased from Sigma Chemical, St. Louis, MO, USA.

### Cell culture

Human gastric adenocarcinoma SGC7901 cells were cultured in the central laboratory of Shengjing Hospital Affiliated to China Medical University [16], and adriamycin-resistant SGC7901/ADR cells were gifted from Institute of Digestive Diseases in Xijing Hospital Affiliated to Fourth Military Medical University [17]. Human gastric adenocarcinoma SGC7901 cells and adriamycin-resistant SGC7901/ADR cells were cultured in RPMI-1640 medium supplemented with 10% FBS and antibiotics (100 U/ml penicillin and streptomycin) with 5% CO_2_ in a humidified incubator at 37°C. ADR (1 µg/ml) was added to the SGC7901/ADR cell culture medium to help maintain their MDR. Cells in the logarithmic growth phase were centrifuged and diluted with RPMI-1640 medium to prepare single-cell suspensions with a concentration of 2.5×10^6^/ml for seeding.

### Establishment of a nude mice xenograft model

The in vivo antitumor effect of PAB was examined in a murine xenograft model. For the model, 4-6-week-old male and female immunodeficient BALB/c (nu/nu) mice, weighing 18 to 22 g, were purchased from Beijing HFK Bioscience Co., Ltd. (China) and kept in accredited facilities under standard conditions for rodents (SPF grade) in the Department of Laboratory Animal Science. Fifty mice were selected and randomly assigned into two groups (25 per group). A total of 25 mice were subcutaneously injected with 2.5×10^6^/ml SGC7901 cells in 0.2 ml of RPMI-1640 medium into the left axillae, and the other 25 mice were injected with SGC7901/ADR cells into the right axillary region under germ-free conditions.

### Experimental groups and medication

Seven days after cell implantation, the tumors became palpable (approximately 3 mm×3 mm in diameter), and then, the two groups injected with two different types of cells were randomly divided into five subgroups (5 mice per group): normal saline (NS) control group, TWEEN control group, ADR group, PAB group, and PAB+ADR group. For the PAB groups, PAB (25 mg/kg/d in 0.1 ml) dissolved in an aqueous solution of 6% polyethylene glycol, 3% ethanol, and 1% Tween80 was administered intraperitoneally (i.p.) daily for 20 days [13]. An identical volume of aqueous solution or NS was injected in the TWEEN control group or NS control group, respectively. For the ADR group, ADR (1.25 mg/kg in 0.1 ml) diluted in NS was administered i.p. daily for 20 days [18]. Tumor-bearing mice were administered both PAB (25 mg/kg/d) and ADR (1.25 mg/kg/day) i.p. for the same period in the PAB+ADR group. After treatment, mice from each group were sacrificed, and tumor samples of bilateral axillary regions were weighed and resected for immunohistochemical and western blot analyses. This study was performed strictly in accordance with the recommendations in the Guide for the Care and Use of Laboratory Animals of the National Institutes of Health. The protocol was approved by the Committee on the Ethics of Animal Experiments of the Shengjing Hospital Affiliated to China Medical University (Permit Number: 2013PS144K). Mice were sacrificed under 10% chloral hydrate anesthesia, and all efforts were made to minimize suffering.

### Measurement and calculation of tumor volume and weight

Body weight was monitored every day, and two perpendicular diameters (length and width in millimeters) of tumors were measured every two days with calipers throughout the treatment period. Tumor volumes were estimated using two-dimensional measurements and calculated as follows: tumor volume (mm^3^)  =  length (mm)×width^2^ (mm)/2. The relative tumor volume (RTV) was calculated as follows: RTV  =  (mean tumor volume during therapy)/(mean tumor volume at the beginning of therapy). The antitumor effects of PAB and/or ADR were estimated with the mean inhibition rate (IR) of tumor growth using the following equation: IR (%)  =  [(1-mean RTV of drug-treated group/mean RTV of NS control group) ×100] [19]. The relative body weight of mice was calculated using the W_t_/W_0_ index, where W_t_ was the body weight on the day of measurement, and W_0_ was the body weight at the first day of treatment. The change in body weight on days 1 and 20, representing the start and end of the experiment, respectively, was used to evaluate and analyze the effects of drugs on the body weight [20].

### Immunohistochemical staining for expression of Cox-2, PKC-α and P-gp

Tumor specimens were fixed with paraformaldehyde, processed routinely by embedding in paraffin, and then sliced into 4-µm sections. Following hematoxylin and eosin (HE) staining, cells of the gastric cancer xenografts were observed under a light microscope. The expression levels of Cox-2, PKC-α and P-gp in harvested tumor cells were determined using the SP method. Briefly, after being dewaxed and rehydrated, slides were incubated in 0.3% H_2_O_2_ solution and dried in a microwave in citric acid buffer for 15 min to retrieve antigens. The slices were blocked with normal goat serum for 30 min at 37°C and probed with a rabbit monoclonal antibody to Cox-2 and mouse monoclonal antibodies to PKC-α and P-gp at 4°C overnight (all antibodies were diluted at 1∶300). Biotinylated anti-rabbit IgG and anti-rat IgG were added and incubated at 37°C for 30 min before enzyme conjugated HRP-streptavidin was added. In addition, 3, 3’-diaminobenzidine (DAB) was used as the chromogen. Slides were counterstained with hematoxylin, dehydrated in alcohol, and mounted on neutral balsam. Controls were prepared using secondary antibodies only. Five fields of each section from every mouse in each group were selected for image collection. The results were analyzed and compared statistically using NIS-Elements Br 3.0 software, and the average optical density was obtained to quantify the expression levels of Cox-2, PKC-α and P-gp.

### Western blot of Cox-2, PKC-α and P-gp in nude mice xenografts

A total of 100 mg of fresh-frozen tumor tissues from at least three mice of each group were weighed and supplemented with radio immunoprecipitation assay (RIPA) lysis buffer [containing 100 µg/ml phenylmethanesulfonyl fluoride (PMSF)]. After pulverized using scissors, extracts were homogenized using ultrasound. After centrifuging at 14000 rpm at 4°C for 30 min, a supernatant sample was frozen at −80°C for western blotting. The protein concentration of the extracts was determined using a bicinchoninic acid (BCA) protein assay kit. A total of 100 µg protein per mouse under denaturing conditions (100°C, 5 min) was electrophoresed using sodium dodecyl sulfate-polyacrylamide gel electrophoresis (SDS-PAGE) on 8% separation gel and electrotransferred onto PVDF membranes using a wet transfer system at 100 V (Cox-2, PKC-α, β-actin for 100 min, and P-gp for 3 h). After blocking with 5% nonfat dry milk in 1×TBST (Tris-buffered saline containing 0.1% Tween20) for 90 min, the membranes were then incubated with the primary antibody [rabbit monoclonal antibody to Cox-2 (1∶400) or mouse monoclonal antibodies to PKC-α (1∶200) and P-gp (1∶200), with β-actin as an internal control for protein loading] overnight at 4°C. The antibody binding was visualized using a peroxidase-coupled secondary goat anti-rabbit antibody (1∶2000) and goat anti-mouse antibody (1∶2000) for 1.5 h at room temperature. Then, the membranes were visualized by enhanced chemiluminescence (ECL) reagent. The results were analyzed using Image J software.

### Statistical analysis

All data are presented as the mean values±standard deviation, and multiple comparisons between any two of the treated groups were evaluated by one-way ANOVA, using SNK and LSD methods with SPSS 17.0 software. The relationship between body weight change and tumor volume were analyzed by Pearson correlation analysis. p values less than 0.05 were considered to be statistically significant.

## Results

### Inhibitory effect of PAB on the growth of human gastric cancer

**Figure 2:**
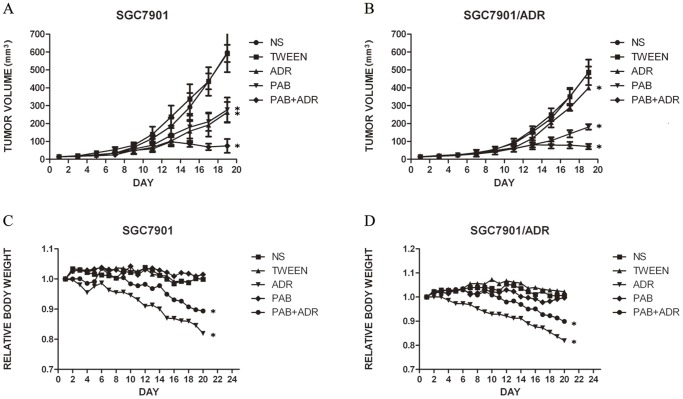
Inhibition of gastric tumor growth by pseudolaric acid B and mean body weight change of nude mice. A: growth curve of SGC7901 cell xenografts B: growth curve of SGC7901/ADR cell xenografts C: relative body weight change of mice with SGC7901 cell xenografts D: relative body weight change of mice with SGC7901/ADR cell xenografts. SGC7901 cells and SGC7901/ADR cells (2.5×10^6^/ml) were injected subcutaneously into the axillary areas of nude mice, and when evident tumors were observed, the mice received a daily dose of 25 mg/kg pseudolaric acid B and/or 1.25 mg/kg adriamycin or normal saline (control)/tween solution (control) (n = 5 per group). Tumor volumes were monitored at different time points (Day1, 3, 5, 7, 9, 11, 13, 15, 17, 19). The IR of tumor growth in the drug-treated groups were compared with that in the control groups at the end of the experiment by one-way ANOVA. *p<0.05 vs control group.

**Figure 3:**
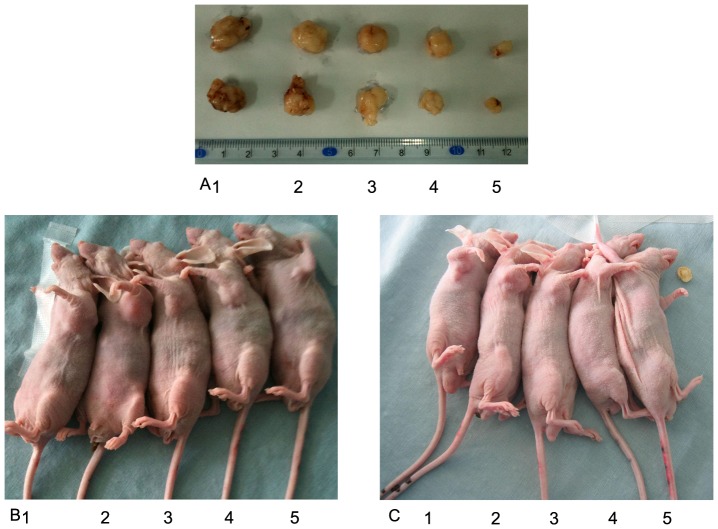
Subcutaneous xenografts of nude mice in different treatment groups. A: isolated tumors of SGC7901 cells (above) and SGC7901/ADR cells (below) B: subcutaneous xenografts of SGC7901 cell line in nude mice C: subcutaneous xenografts of SGC7901/ADR cell line in nude mice. (group 1): NS control group (group 2): TWEEN control group (group 3): ADR group (group 4): PAB group (group 5): PAB+ADR group.

Tumors in all treated groups formed readily after the implantation of single-cell suspensions, and the formation rate was 100%. When the xenografts became evident, there were no significant differences in the average volume of the xenografts among the different groups (Table 1), and the tumor volumes were monitored at various time points in all groups. The curves of the tumor growth of the two cell lines were graphed (Figure 2A, 2B). As shown in Table 1, for the SGC7901 cell-treated groups, no significant difference was observed between the PAB group and ADR group in the average relative volumes of the xenografts (p>0.05), and the IR was 56.4% and 59.0%, respectively. The addition of ADR to PAB resulted in higher antitumor activity than that of PAB alone or ADR alone (p<0.05) with an IR of 88.1%. The inhibitory effect of the PAB group was stronger compared with that of the ADR group on the xenografts of SGC7901/ADR cells, and the IR values were 64.1% and 21.9%, respectively (p<0.05). Tumor growth inhibition was more evident in mice treated with PAB combined with ADR, with an IR of 85.8%. The findings of tumor weight in different groups further supported our results regarding the volume of mice xenografts. Figure 2C and 2D shows the changes of the relative body weight of the mice in the ten groups. The body weight was similar between the control groups and PAB-treated groups. However, the body weight decreased in the combination-treated groups and ADR groups, and the decrease of the ADR groups was more evident (p<0.05). Moreover, Figure 3 provides a direct comparison of the tumor sizes of the SGC7901 cells and SGC7901/ADR cells in the different groups. The relationship between body weight change and tumor volume were not significant (p>0.05). The dosages tested were well tolerated by the mice, and no animal lethality was observed during the experiment.

**Table 1:** Antitumor effects of PAB and/or ADR on nude mice xenografts of SGC7901 cells and SGC7901/ADR cells.

	Number of animals	Body weight (g)	Volume of tumors (mm^3^)	Relative tumor volume (mm^3^)	Rtv/Ctv (%)	Tumor weight (g)	Inhibition rate (%)	Body weight change (%)
	Beginning Ending	Beginning Ending	Beginning Ending					
**SGC7901**								
NS control group	5 5	21.3±2.8 21.2±2.0	13.5±2.5 596.8±109.6	44.3	100	1.05±0.09	0	−5.11±3.8
TWEEN control group	5 5	20.7±2.5 20.7±2.6	13.7±0.5 592.1±48.4	43.2	97.6	1.05±0.08	2.4	−5.13±2.9
ADR group	5 5	22.6±2.6 18.5±2.1	14.5±4.0 262.7±57.9	18.2	41.0	0.44±0.06*	59.0	−20.1±2.9
PAB group	5 5	20.7±3.5 21.0±3.7	14.4±2.1 276.8±68.6	19.3	43.6	0.49±0.1*	56.4	−0.98±6.0
PAB+ADR group	5 5	21.3±2.1 19.0±1.6	14.2±1.7 74.8±38.7	5.3	12.0	0.15±0.0*	88.1	−11.3±3.8
**SGC7901/ADR**								
NS control group	5 5	21.1±2.5 21.2±2.3	13.6±0.7 486.7±31.4	35.8	100	0.84±0.06	0	−3.44±2.4
TWEEN control group	5 5	20.7±2.3 21.1±2.0	13.0±1.3 486.2±71.3	37.4	104.5	0.83±0.12	−4.5	−1.90±5.2
ADR group	5 5	22.7±1.0 18.6±1.7	14.3±2.0 399.3±12.6	28.0	78.1	0.69±0.03*	21.9	−21.2±5.9
PAB group	5 5	21.1±2.5 21.0±2.2	14.1±2.0 180.8±15.6	12.8	35.9	0.32±0.03*	64.1	−1.87±2.2
PAB+ADR group	5 5	21.2±2.3 19.0±1.4	13.8±1.5 71.3±15.0	5.1	14. 2	0.13±0.04*	85.8	−10.7±3.7

Notes: * p<0.05 vs control group.

Rtv/Ctv (%)  =  (Relative tumor volume in treatment group/Relative tumor volume in NS control group) ×100.

Inhibition rate (%)  =  [(1-mean RTV of drug-treated group/mean RTV of NS control group) ×100].

Body weight (BW) change (%)  =  [(BW on day 20- tumor weight) - (BW on day 1)]/(BW on day 1) ×100.

PAB, Pseudolaric acid B; ADR, adriamycin.

### Immunohistochemical staining for the expression of Cox-2, PKC-α and P-gp

**Figure 4:**
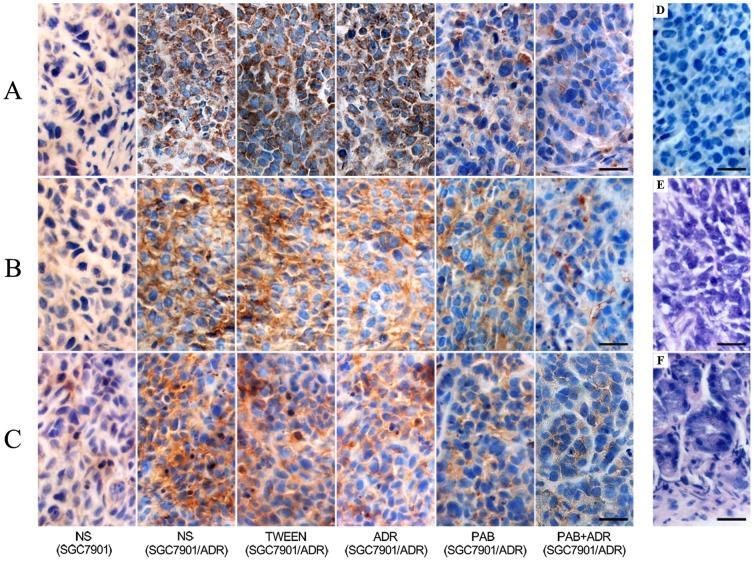
Cox-2, PKC-α, and P-gp expression levels of xenografts in different groups (×400). A: expression of Cox-2 in mice xenografts B: expression of PKC-α in mice xenografts C: expression of P-gp in mice xenografts D: negative control E: xenograft by HE F: normal gastric tissue by HE. Tissues were fixed, embedded, mounted and stained by the SP method or HE staining using standard procedures. Scale bar, 25 µm.

All tumors in each group, via immunohistochemical staining, displayed positive expression of the Cox-2, PKC-α and P-gp proteins. Cox-2 and PKC-α expression was defined as positive if the stained region of the tumor cells was in the cytoplasm, and P-gp staining was considered positive if staining was observed in the membrane and cytoplasm. The proteins were stained yellow or brown under optical microscope observation (Figure 4). For the SGC7901/ADR cell line, the staining of these proteins in the control groups was stronger than in the other groups (p<0.05). PAB treatment reduced the intracellular staining of Cox-2, PKC-α and P-gp compared with the ADR-treated group (p<0.05). After PAB and ADR treatment, the intracellular staining of these proteins was sparser and weaker compared with that of the other groups. For the xenografts of the two NS control groups, the staining of P-gp in the SGC7901 cells was significantly lower than that of the SGC7901/ADR cells (p<0.05). The analysis of the staining intensity of Cox-2, PKC-α and P-gp expression revealed the same results (Table 2).

**Table 2:** Average optical density of Cox-2, PKC-α and P-gp in different treatment groups (mean ± SD).

Group	n	Cox-2	PKC-α	P-gp
NS control group (SGC7901)	5	0.243±0.011*	0.192±0.012*	0.219±0.008*
NS control group (SGC7901/ADR)	5	0.302±0.004	0.285±0.006	0.292±0.008
TWEEN control group (SGC7901/ADR)	5	0.304±0.007	0.287±0.007	0.291±0.010
ADR group (SGC7901/ADR)	5	0.291±0.007*	0.252±0.002*	0.261±0.005*
PAB group (SGC7901/ADR)	5	0.255±0.004*	0.222±0.002*	0.241±0.005*
PAB+ADR group (SGC7901/ADR)	5	0.236±0.002*	0.183±0.009*	0.226±0.003*

Notes: * p<0.05 vs control group.

n: number of animals.

PAB, Pseudolaric acid B; ADR, adriamycin; SD, standard deviation.

### Effect of PAB on Cox-2, PKC-α, and P-gp expression in mice xenografts

**Figure 5:**
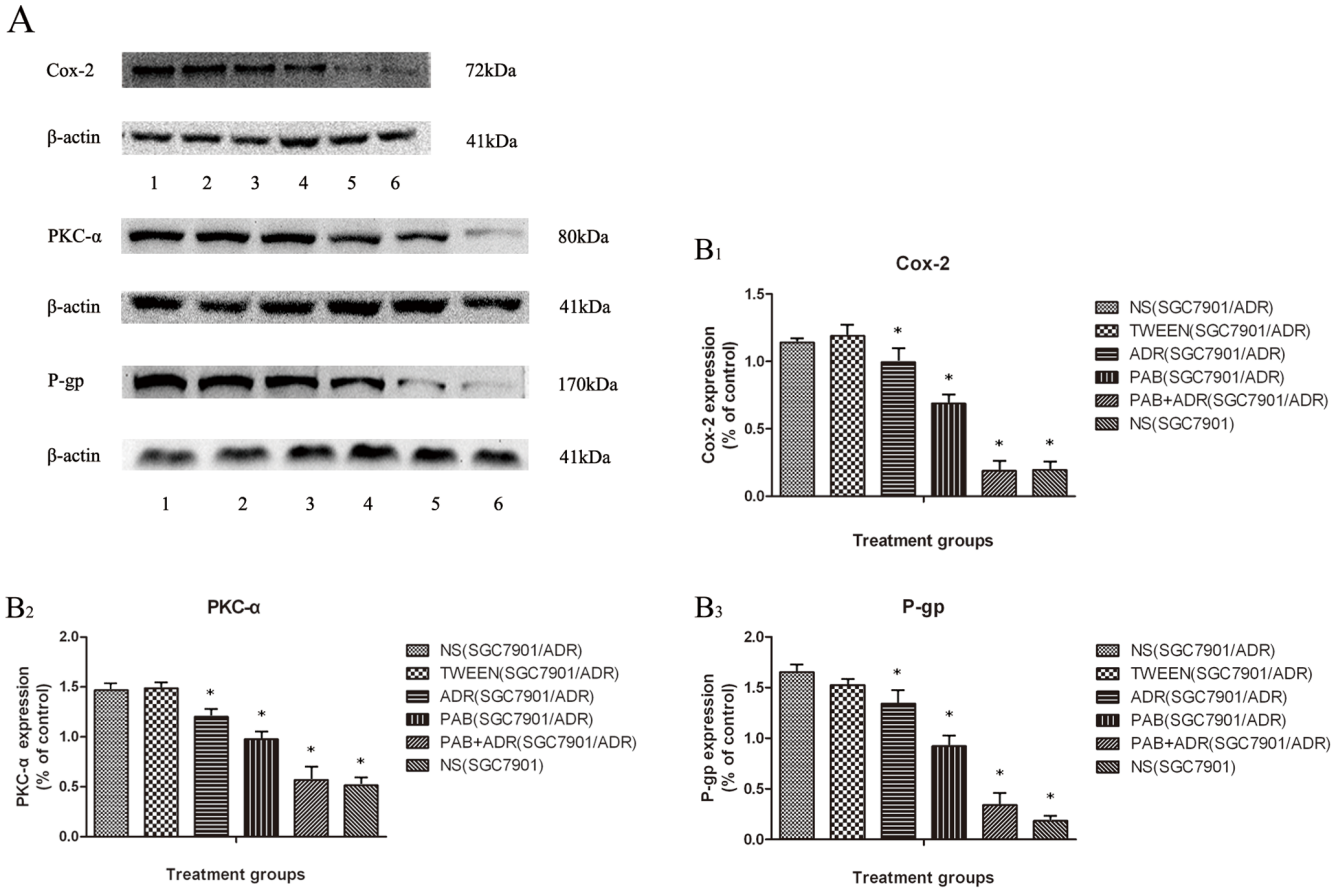
Inhibitory effects of PAB on expressions of Cox-2, PKC-α and P-gp in tumor tissues. A: representative immunoblots of Cox-2, PKC-α, P-gp compared with β-actin in different treatment groups. (lane 1): NS control group (SGC7901/ADR) (lane 2): TWEEN control group (SGC7901/ADR) (lane 3): ADR group (SGC7901/ADR) (lane 4): PAB group (SGC7901/ADR) (lane 5): PAB+ADR group (SGC7901/ADR) (lane 6): NS control group (SGC7901). B: relative expression levels of Cox-2 (B1), PKC-α (B2), and P-gp (B3) in different treatment groups. The tumors were isolated and homogenized to measure the protein level, and β-actin was used as an internal control. Each immunoblot was representative of three distinct experiments with similar results. *p<0.05 vs control group.

To elucidate the molecular mechanism involved in the reversal effects of PAB on the SGC7901/ADR xenografts in vivo, the expression of Cox-2, PKC-α, and P-gp in tumors from different reagent-treated groups were determined by western blot analysis. As shown in Figure 5, for the NS control group, the expression levels of Cox-2, PKC-α, and P-gp in the SGC7901 cell xenografts were lower compared than those in the SGC7901/ADR cell tumors (p<0.05). For the tumors of the SGC7901/ADR cells, the expression levels of the three proteins of interest in the NS control group and TWEEN control group were significantly higher compared with the other groups (p<0.05), and there was no significant difference between the two groups (p>0.05). The expression levels of Cox-2, PKC-α, and P-gp in the PAB-treated group were significantly lower compared with those of the ADR-treated group (p<0.05). The expression of β-actin was used as an internal control, and the expression levels of Cox-2, PKC-α, and P-gp were decreased sequentially among the control groups, ADR group, PAB group and PAB+ADR group (p<0.05).

## Discussion

PAB is one of the major biologically active components of the root bark of the medicinal plant *Pseudolarix kaempferi* and displays considerable cytotoxicity toward several cancer cell lines. In vitro studies have also demonstrated that it reverses the MDR of carcinoma by inhibiting the overexpression of P-gp without obvious side effects [13], [15], [20], [21]. It has also been demonstrated that PAB has an antitumor effect in mice xenograft models of human colon cancer and hepatocellular carcinoma [13], [20], and no known side effects of PAB on animals have been reported. Our previous study demonstrated that PAB has a potent inhibitory effect on the SGC7901 cell line in vitro and induces apoptosis of gastric cancer cells via the caspase pathway [16]. Our present in vivo study provides further evidence that PAB is protective against gastric tumors in murine xenograft models using SGC7901 cells and SGC7901/ADR cells. Our preliminary experiments have shown that PAB at 25 mg/kg not only inhibits the growth of gastric cancer but is also well tolerated by mice. Moreover, we included the TWEEN control group to exclude the influence of the aqueous solution that was used to dissolve PAB on tumors [13]. For sensitive cells, the anticancer effects of PAB produced no significant differences compared with ADR, a traditional chemotherapy drug. In addition, PAB is a biologically active compound that exerted its reversing effect against SGC7901/ADR cell line tumors by targeting the signaling pathway activated by P-gp overexpression in vivo.

P-gp is a 170-kDa membrane protein that acts as a drug efflux pump, resulting in a continuous defect in the intracellular accumulation of drugs [22]. The multidrug resistance phenotype is the major cause of tumor chemotherapy failure and is mainly the product of the overexpression of P-gp in the majority of cancers, including gastric neoplasms [23]. Moreover, the inhibition of this pathway might be a way to reverse the MDR of gastric cancer cells [3], [24].

Cox-2, an inducible enzyme that catalyzes the conversion of arachidonic acid to prostanoids (PG) and participates in multiple physiological and pathological events, is induced by various inflammatory and mitogenic stimuli [25]. A strong association between MDR and the overexpression of Cox-2 has been reported in many different types of tumors. These previous results suggest that Cox-2 modulates the expression and activity of P-gp and is involved in the development of the MDR phenotype [26]–[28]. Moreover, accumulated evidence has demonstrated that Cox-2 inhibitors are able to sensitize cancer cells to the anti-proliferative effects of chemotherapeutic drugs by altering the activity of the ATP-binding cassette protein. Cox-2 inhibitors can even reverse MDR by blocking the Cox-2-mediated increase in MDR1 expression and activity in cancer, both in vitro and in vivo [6], [7], [29]. Therefore, we hypothesize that Cox-2 may regulate the expression of P-gp in tumor tissues and that inhibiting this pathway is of great significance to reversing the resistance of cancer to chemotherapeutic drugs.

We found that for the SGC7901/ADR cell tumors, the expression levels of Cox-2 and P-gp in the NS control group were evidently higher than those for the xenografts of SGC7901 cells. After the injection of PAB, an obvious decrease in these expression levels was observed. However, ADR was less effective for the suppression of the Cox-2 and P-gp expression levels of the SGC7901/ADR cell xenografts, and when combined with PAB, a lower level of proteins was observed. Thus, we proposed that modulation of Cox-2 expression by PAB could decrease the expression and activity of P-gp, inhibit growth and induce apoptosis, in addition to acting cooperatively with ADR in the suppression of drug-resistant tumors and even the reversal of the MDR phenotype of gastric cancer.

PKC is a serine/threonine kinase involved in the signal transduction required for cellular proliferation and differentiation [30]. Of the PKC isoforms, the overexpression and increased activity of PKC-α are most closely associated with the regulation of the MDR phenotype in human gastric cancer [31]. The results presented here support the notion that PKC-α phosphorylates P-gp, modulates P-gp efflux function, and ultimately leads to drug resistance [32]. The inhibition of PKC-α with a specific inhibitor or knocking down PKC-α with siRNA leads to reduced MDR1 expression, increased toxicity of anticancer drugs, and reversed MDR [8]–[10]. Therefore, we speculate that a PKC-α signal transduction system might play a role in modulating MDR1 expression in gastric cancer.

Under normal circumstances, PKC is inactive in cells, and its activation is related to upstream PGE activation. Cox-2 is coupled to membrane-associated prostaglandin E2 synthase (MPGES-1), and synthesis of PGE mediated by these proteins increases and activates the downstream PKC-α pathway [33]. It has been demonstrated that selective Cox-2 inhibition by specific inhibitors or siRNA has a therapeutic effect on inhibiting the expression of PKC-α and P-gp, while also, at the same time, reversing MDR [26], [34]. A study has demonstrated that PGE2 mediates induction of MDR1 expression via the PKC pathway [35]. The results of our experiments demonstrated that PAB can inhibit PKC-α expression of xenografts of the SGC7901/ADR cell line, the effect of which was stronger than that of ADR. In addition, when PAB was combined with ADR, the PKC-α expression inhibition efficacy was even stronger. The variation tendency of PKC-α expression is consistent with that of Cox-2 and P-gp in various reagent-treated groups of drug-resistant SGC7901/ADR tumors. It has been shown that PAB induces growth arrest and apoptosis through inhibiting the Cox-2 pathway in cancer cells [20], [21]. We hypothesize that PAB inhibits the expression of Cox-2 in SGC7901/ADR cell xenografts and that the coupling between Cox-2 and MPGES-1 reduces the synthesis of PGE, which reduces the activation of downstream PKC-α. As the phosphorylation of P-gp is inhibited, more drugs accumulate in the cancer cells, the toxicity of ADR is enhanced and MDR is reversed. These results provide insight into a new strategy involving the use of PAB to inhibit MDR of human gastric cancers.

One focus of our experiment is the inhibitory effect of pseudolaric acid B on subcutaneous xenografts of human gastric adenocarcinoma and the comparison of anti-tumor effects of drugs among different treatment groups. Moreover, the characteristic of SGC7901 cells and SGC7901/ADR cells are different. Through our two pre-experiment and a formal experiment, we all found the tumor volume of SGC7901/ADR cells was not larger than that of SGC7901 cells. We hypothesize the tumor volume might be associated with the cell count, growth time, characteristic of cells and other kinds of factors. Therefore, we have not compared the tumor volume between SGC7901 cell xenografts and SGC7901/ADR cell xenografts. In addition, we speculate that the body weight change of mice might be mainly related to the toxicity of drugs, and have not significant relationship to the tumor volume in our experiment.

The other focus of our experiment is the underlying molecular mechanisms involved in the multidrug resistance. Thus we have only designed NS control group of SGC7901 cell xenografts to contrast and confirm the expressions of the three kinds of protein from SGC7901/ADR cell xenografts are indeed significantly higher than that from SGC7901 cell xenografts. Besides, since the response of SGC7901 cells to anticancer drugs is rather more sensitive than SGC7901/ADR cells in both basic and clinical experiments, how to improve the sensitivity of multidrug resistance cells to anticancer drugs is our research focus. Therefore, we have not compared the expressions of Cox-2, PKC-α and P-gp for the tumors from SGC7901 and the various treatments of this group except for NS control group by western blot and immunohistochemical staining.

Our study discovered that the herbal diterpenoid PAB not only significantly suppressed gastric cancer SGC7901 cells in vivo but also displayed obvious inhibitory effects toward tumors of drug-resistant SGC7901/ADR cells. Moreover, the combination of PAB with ADR could inhibit the growth of gastric cancer and the development of xenografts in nude mice. It is worth mentioning that as a type of traditional Chinese medicine, the effect of PAB on the body weight of nude mice was quite lower than that of ADR in our experiment, which is an apparent advantage that makes PAB a more secure and tolerable drug for cancer therapy. PAB might not only be a new effective drug for the inhibition of the growth of gastric cancer and reversing its MDR, but it also can be used as an excellent adjuvant with traditional chemotherapeutics and surgical therapy following further development and research.

In the present study, we examined, for the first time, the inhibitory effect of PAB against human gastric cancer and its reversal effect on MDR in vivo via a xenograft nude mice model. We determined that its inhibition of MDR was mediated through the Cox-2/PKC-α/P-gp pathway, which provides a crucial theoretical basis concerning the molecular mechanisms of gastric cancer therapy. However, we have not studied the MDR mechanism in regards to gene expression and regulation. As the molecular mechanisms of reversing MDR are extraordinarily complicated and multiple types of proteins, genes and factors may be involved, the loop that we have proposed may be only a part of the mechanism for reversing MDR. More in-depth and concrete regulation mechanisms need to be elucidated in future studies.

In conclusion, PAB has a significant inhibitory effect on gastric cancer in vivo and can reverse the MDR of gastric cancer to chemotherapy drugs. The mechanism against MDR is mediated at least partially through the Cox-2/PKC-α/P-gp pathway. Our study provides broad prospects for future investigations of PAB in gastric cancer therapy.

## Supporting Information

Checklist S1 **ARRIVE Guidelines Checklist.**
(DOC)
