# Supplementary material for: The Inhibitory Effect of Pseudolaric Acid B on Gastric Cancer and Multidrug Resistance via Cox-2/PKC-α/P-gp Pathway
Source: PLoS One. 2014 Sep 24;9(9):e107830. doi: 10.1371/journal.pone.0107830 (PMC4176716; doi:10.1371/journal.pone.0107830)
Supplement: Checklist S1 — ARRIVE Guidelines Checklist. (DOC) [file pone.0107830.s001.doc]

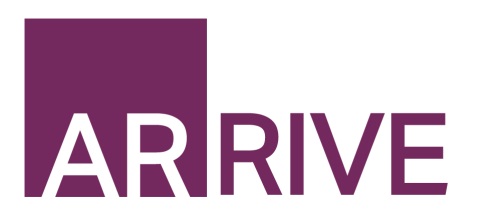


The ARRIVE Guidelines Checklist

Animal Research: Reporting In Vivo Experiments

Carol Kilkenny^1^, William J Browne^2^, Innes C Cuthill^3^, Michael Emerson^4^ and Douglas G Altman^5^

*^1^The National Centre for the Replacement, Refinement and Reduction of Animals in Research, London, UK, ^2^School of Veterinary Science, University of Bristol, Bristol, UK, ^3^School of Biological Sciences, University of Bristol, Bristol, UK, ^4^National Heart and Lung Institute, Imperial College London, UK, ^5^Centre for Statistics in Medicine, University of Oxford, Oxford, UK.*

|  | ITEM | RECOMMENDATION | Section/ Paragraph |
| --- | --- | --- | --- |
| Title | 1 | Provide as accurate and concise a description of the content of the article as possible. | The inhibitory effect of pseudolaric acid B on gastric cancer in vivo |
| Abstract | 2 | Provide an accurate summary of the background, research objectives, including details of the species or strain of animal used, key methods, principal findings and conclusions of the study. | Human gastric adenocarcinoma SGC7901 cells and drug-resistant SGC7901/ADR cells were injected into nude mice to establish a subcutaneous xenograft model. The effects of pseudolaric acid B with or without adriamycin treatment were compared by determining the tumor size and weight. Pseudolaric acid B has a significant inhibitory effect and an additive inhibitory effect in combination with adriamycin on the growth of gastric cancer in vivo. The aim of the present study is to evaluate the anti-neoplastic effect of PAB in vivo, including the reversal of MDR, using a xenograft model in nude mice |
| INTRODUCTION | | |  |
| Background | 3 | a. Include sufficient scientific background (including relevant references to previous work) to understand the motivation and context for the study, and explain the experimental approach and rationale.  b. Explain how and why the animal species and model being used can address the scientific objectives and, where appropriate, the study’s relevance to human biology. | In vitro studies of the anticancer effect of PAB are rare, and neither the in vivo efficacy of this novel herbal compound against tumors nor its precise molecular mechanism against MDR have been fully investigated. |
| Objectives | 4 | Clearly describe the primary and any secondary objectives of the study, or specific hypotheses being tested. | The aim of the present study is to evaluate the anti-neoplastic effect of PAB in vivo, including the reversal of MDR, using a xenograft model in nude mice and explore whether PAB’s underlying molecular mechanism is the inhibition of MDR via the Cox-2/PKC-α/P-gp pathway. |
| METHODS | | |  |
| Ethical statement | 5 | Indicate the nature of the ethical review permissions, relevant licences (e.g. Animal [Scientific Procedures] Act 1986), and national or institutional guidelines for the care and use of animals, that cover the research. | This study was performed strictly in accordance with the recommendations in the Guide for the Care and Use of Laboratory Animals of the National Institutes of Health. The protocol was approved by the Committee on the Ethics of Animal Experiments of the Shengjing Hospital Affiliated to China Medical University (Permit Number: 2013PS144K). Mice were sacrificed under 10% chloral hydrate anesthesia, and all efforts were made to minimize suffering. |
| Study design | 6 | For each experiment, give brief details of the study design including:  a. The number of experimental and control groups.  b. Any steps taken to minimise the effects of subjective bias when allocating animals to treatment (e.g. randomisation procedure) and when assessing results (e.g. if done, describe who was blinded and when).  c. The experimental unit (e.g. a single animal, group or cage of animals).  A time-line diagram or flow chart can be useful to illustrate how complex study designs were carried out. | Fifty mice were selected and randomly assigned into two groups (25 per group). Two kinds of cells were subcutaneously injected into the axillaes to establish the nude mice xenograft model of gastric cancer. Then, the two groups were randomly divided into five subgroups (5 mice per group): normal saline (NS) control group, TWEEN control group, ADR group, PAB group, and PAB+ADR group. Relevant reagents were administered intraperitoneally (i.p.) daily for 20 days.The antitumor effects of PAB and/or ADR were estimated with the volume and weight of xenografts, and the body weigh change was used to evaluate the effect of drugs on body weight of mice. |
| Experimental procedures | 7 | For each experiment and each experimental group, including controls, provide precise details of all procedures carried out. For example:  a. How (e.g. drug formulation and dose, site and route of administration, anaesthesia and analgesia used [including monitoring], surgical procedure, method of euthanasia). Provide details of any specialist equipment used, including supplier(s).  b. When (e.g. time of day).  c. Where (e.g. home cage, laboratory, water maze).  d. Why (e.g. rationale for choice of specific anaesthetic, route of administration, drug dose used). | 4-6-week-old male and female immunodeficient BALB/c (nu/nu) mice, weighing 18 to 22 g, were kept in accredited facilities under standard conditions for rodents (SPF grade). Fifty mice were selected and randomly assigned into two groups (25 per group). A total of 25 mice were subcutaneously injected with 2.5×10^6^/ml SGC7901 cells in 0.2 ml of RPMI-1640 medium into the left axillae, and the other 25 mice were injected with SGC7901/ADR cells into the right axillary region under germ-free conditions. Seven days after cell implantation, the tumors became palpable (approximately 3 mm×3 mm in diameter), and then, the two groups injected with two different types of cells were randomly divided into five subgroups (5 mice per group): normal saline (NS) control group, TWEEN control group, ADR group, PAB group, and PAB+ADR group. For the PAB groups, PAB (25 mg/kg/d in 0.1 ml) dissolved in an aqueous solution of 6% polyethylene glycol, 3% ethanol, and 1% Tween80 was administered intraperitoneally (i.p.) daily for 20 days. An identical volume of aqueous solution or NS was injected in the TWEEN control group or NS control group, respectively. For the ADR group, ADR (1.25 mg/kg in 0.1 ml) diluted in NS was administered i.p. daily for 20 days. Tumor-bearing mice were administered both PAB (25 mg/kg/d) and ADR (1.25 mg/kg/day) i.p. for the same period in the PAB+ADR group. After treatment, mice from each group were sacrificed, and tumor samples of bilateral axillary regions were weighed and resected for immunohistochemical and western blot analyses. Body weight was monitored every day, and two perpendicular diameters (length and width in millimeters) of tumors were measured every two days with calipers throughout the treatment period. |
| Experimental animals | 8 | a. Provide details of the animals used, including species, strain, sex, developmental stage (e.g. mean or median age plus age range) and weight (e.g. mean or median weight plus weight range).  b. Provide further relevant information such as the source of animals, international strain nomenclature, genetic modification status (e.g. knock-out or transgenic), genotype, health/immune status, drug or test naïve, previous procedures, etc. | For the model, 4-6-week-old male and female immunodeficient BALB/c (nu/nu) mice, weighing 18 to 22 g, were purchased from Beijing HFK Bioscience Co., Ltd. (China) and kept in accredited facilities under standard conditions for rodents (SPF grade) in the Department of Laboratory Animal Science. |

The ARRIVE guidelines. Originally published in *PLoS Biology*, June 2010^1^

| Housing and husbandry | 9 | | Provide details of:  a. Housing (type of facility e.g. specific pathogen free [SPF]; type of cage or housing; bedding material; number of cage companions; tank shape and material etc. for fish).  b. Husbandry conditions (e.g. breeding programme, light/dark cycle, temperature, quality of water etc for fish, type of food, access to food and water, environmental enrichment).  c. Welfare-related assessments and interventions that were carried out prior to, during, or after the experiment. | Mice were kept in accredited facilities under standard conditions for rodents (SPF grade) in the Department of Laboratory Animal Science with adequate sterilized water intake and feed for the animals free. | |
| --- | --- | --- | --- | --- | --- |
| Sample size | 10 | | a. Specify the total number of animals used in each experiment, and the number of animals in each experimental group.  b. Explain how the number of animals was arrived at. Provide details of any sample size calculation used.  c. Indicate the number of independent replications of each experiment, if relevant. | 4-6-week-old male and female immunodeficient BALB/c (nu/nu) mice, weighing 18 to 22 g, were purchased from Beijing HFK Bioscience Co., Ltd. (China) and kept in accredited facilities under standard conditions for rodents (SPF grade) in the Department of Laboratory Animal Science. The dosages tested were well tolerated by the mice, and no animal lethality was observed during the experiment | |
| Allocating animals to experimental groups | 11 | | a. Give full details of how animals were allocated to experimental groups, including randomisation or matching if done.  b. Describe the order in which the animals in the different experimental groups were treated and assessed. | Fifty mice were selected and randomly assigned into two groups (25 per group). A total of 25 mice were subcutaneously injected with 2.5×10^6^/ml SGC7901 cells in 0.2 ml of RPMI-1640 medium into the left axillae, and the other 25 mice were injected with SGC7901/ADR cells into the right axillary region under germ-free conditions. Seven days after cell implantation, the tumors became palpable (approximately 3 mm×3 mm in diameter), and then, the two groups injected with two different types of cells were randomly divided into five subgroups (5 mice per group): normal saline (NS) control group, TWEEN control group, ADR group, PAB group, and PAB+ADR group. For the PAB groups, PAB (25 mg/kg/d in 0.1 ml) dissolved in an aqueous solution of 6% polyethylene glycol, 3% ethanol, and 1% Tween80 was administered intraperitoneally (i.p.) daily for 20 days. An identical volume of aqueous solution or NS was injected in the TWEEN control group or NS control group, respectively. For the ADR group, ADR (1.25 mg/kg in 0.1 ml) diluted in NS was administered i.p. daily for 20 days . Tumor-bearing mice were administered both PAB (25 mg/kg/d) and ADR (1.25 mg/kg/day) i.p. for the same period in the PAB+ADR group. | |
| Experimental outcomes | 12 | | Clearly define the primary and secondary experimental outcomes assessed (e.g. cell death, molecular markers, behavioural changes). | Pseudolaric acid B significantly suppressed the tumor growth induced by SGC7901 cells and SGC7901/ADR cells. The combination of pseudolaric acid B and the traditional chemotherapy drug adriamycin exhibited more potent inhibitory effects on the growth of gastric cancer in vivo than treatment with either pseudolaric acid B or adriamycin alone. Protein expression levels of cyclo-oxygenase-2, protein kinaseC-α and P-glycoprotein were inhibited by pseudolaric acid B alone or in combination with adriamycin in SGC7901/ADR cell xenografts. | |
| Statistical methods | 13 | | a. Provide details of the statistical methods used for each analysis.  b. Specify the unit of analysis for each dataset (e.g. single animal, group of animals, single neuron).  c. Describe any methods used to assess whether the data met the assumptions of the statistical approach. | All data are presented as the mean values±standard deviation, and multiple comparisons between any two of the treated groups were evaluated by one-way ANOVA, using SNK and LSD methods with SPSS 17.0 software. p values less than 0.05 were considered to be statistically significant. | |
| RESULTS | | | |  | |
| Baseline data | 14 | | For each experimental group, report relevant characteristics and health status of animals (e.g. weight, microbiological status, and drug or test naïve) prior to treatment or testing. (This information can often be tabulated). | For the SGC7901 cell-treated groups, no significant difference was observed between the PAB group and ADR group in the average relative volumes of the xenografts (p>0.05), whereas the IR was 56.4% and 59.0%, respectively. The addition of ADR to PAB resulted in higher antitumor activity than that of PAB alone or ADR alone (p<0.05) with an IR of 88.1%. The inhibitory effect of the PAB group was stronger compared with that of the ADR group on the xenografts of SGC7901/ADR cells, and the IR values were 64.1% and 21.9%, respectively (p<0.05). Tumor growth inhibition was more evident in mice treated with PAB combined with ADR, with an IR of 85.8%. The findings of tumor weight in different groups further supported our results regarding the volume of mice xenografts. Figure 2C and 2D shows the changes of the relative body weight of the mice in the ten groups. The body weight was similar between the control groups and PAB-treated groups. However, the body weight decreased in the combination-treated groups and ADR groups, and the decrease of the ADR groups was more evident (p<0.05). Moreover, Figure 3 provides a direct comparison of the tumor sizes of the SGC7901 cells and SGC7901/ADR cells in the different groups. The dosages tested were well tolerated by the mice, and no animal lethality was observed during the experiment. | |
| Numbers analysed | 15 | | 1. Report the number of animals in each group included in each analysis. Report absolute numbers (e.g. 10/20, not 50%^2^).   b. If any animals or data were not included in the analysis, explain why. | Fifty mice were selected and randomly assigned into two groups (25 per group). for the SGC7901 cell-treated groups, no significant difference was observed between the PAB group and ADR group in the average relative volumes of the xenografts (p>0.05), whereas the IR was 56.4% and 59.0%, respectively. The addition of ADR to PAB resulted in higher antitumor activity than that of PAB alone or ADR alone (p<0.05) with an IR of 88.1%. The inhibitory effect of the PAB group was stronger compared with that of the ADR group on the xenografts of SGC7901/ADR cells, and the IR values were 64.1% and 21.9%, respectively (p<0.05). Tumor growth inhibition was more evident in mice treated with PAB combined with ADR, with an IR of 85.8%. The findings of tumor weight in different groups further supported our results regarding the volume of mice xenografts. Figure 2C and 2D shows the changes of the relative body weight of the mice in the ten groups. The body weight was similar between the control groups and PAB-treated groups. However, the body weight decreased in the combination-treated groups and ADR groups, and the decrease of the ADR groups was more evident (p<0.05). Moreover, Figure 3 provides a direct comparison of the tumor sizes of the SGC7901 cells and SGC7901/ADR cells in the different groups. The dosages tested were well tolerated by the mice, and no animal lethality was observed during the experiment. and then, the two groups injected with two different types of cells were randomly divided into five subgroups (5 mice per group): normal saline (NS) control group, TWEEN control group, ADR group, PAB group, and PAB+ADR group. | |
| Outcomes and estimation | 16 | | Report the results for each analysis carried out, with a measure of precision (e.g. standard error or confidence interval). | For the SGC7901 cell-treated groups, no significant difference was observed between the PAB group and ADR group in the average relative volumes of the xenografts (p>0.05), whereas the IR was 56.4% and 59.0%, respectively. The addition of ADR to PAB resulted in higher antitumor activity than that of PAB alone or ADR alone (p<0.05) with an IR of 88.1%. The inhibitory effect of the PAB group was stronger compared with that of the ADR group on the xenografts of SGC7901/ADR cells, and the IR values were 64.1% and 21.9%, respectively (p<0.05). Tumor growth inhibition was more evident in mice treated with PAB combined with ADR, with an IR of 85.8%. The findings of tumor weight in different groups further supported our results regarding the volume of mice xenografts. Figure 2C and 2D shows the changes of the relative body weight of the mice in the ten groups. The body weight was similar between the control groups and PAB-treated groups. However, the body weight decreased in the combination-treated groups and ADR groups, and the decrease of the ADR groups was more evident (p<0.05). Moreover, Figure 3 provides a direct comparison of the tumor sizes of the SGC7901 cells and SGC7901/ADR cells in the different groups. For sensitive cells, the anticancer effects of PAB produced no significant differences compared with ADR, a traditional chemotherapy drug. In addition, PAB is a biologically active compound that exerted its reversing effect against SGC7901/ADR cell line tumors by targeting the signaling pathway activated by P-gp overexpression in vivo. | |
| Adverse events | 17 | | a. Give details of all important adverse events in each experimental group.  b. Describe any modifications to the experimental protocols made to reduce adverse events. | N/A | |
| DISCUSSION | | | |  | |
| Interpretation/ scientific implications | 18 | | a. Interpret the results, taking into account the study objectives and hypotheses, current theory and other relevant studies in the literature.  b. Comment on the study limitations including any potential sources of bias, any limitations of the animal model, and the imprecision associated with the results^2^.  c. Describe any implications of your experimental methods or findings for the replacement, refinement or reduction (the 3Rs) of the use of animals in research. | PAB has a significant inhibitory effect on gastric cancer in vivo and can reverse the MDR of gastric cancer to chemotherapy drugs. The mechanism against MDR is mediated at least partially through the Cox-2/PKC-α/P-gp pathway.  We examined for the first time the inhibition of pseudolaric acid B (PAB) against human gastric cancer in vivo and its reverse effect on multidrug resistance via xenograft model of nude mice. Further, we supposed that the inhibitory effect of PAB on multidrug resistance was through Cox-2/PKC-α/P-gp pathway. | |
| Generalisability/ translation | 19 | | Comment on whether, and how, the findings of this study are likely to translate to other species or systems, including any relevance to human biology. | In the present study, we examined, for the first time, the inhibitory effect of PAB against human gastric cancer and its reversal effect on MDR in vivo via a xenograft nude mice model. We determined that its inhibition of MDR was mediated through the Cox-2/PKC-α/P-gp pathway, which provides a crucial theoretical basis concerning the molecular mechanisms of gastric cancer therapy. However, we have not studied the MDR mechanism in regards to gene expression and regulation. As the molecular mechanisms of reversing MDR are extraordinarily complicated and multiple types of proteins, genes and factors may be involved, the loop that we have proposed may be only a part of the mechanism for reversing MDR. More in-depth and concrete regulation mechanisms need to be elucidated in future studies.  In conclusion, PAB has a significant inhibitory effect on gastric cancer in vivo and can reverse the MDR of gastric cancer to chemotherapy drugs. The mechanism against MDR is mediated at least partially through the Cox-2/PKC-α/P-gp pathway. Our study provides broad prospects for future investigations of PAB in gastric cancer therapy. | |
| Funding | 20 | List all funding sources (including grant number) and the role of the funder(s) in the study. | | Supported by Science Program Foundation of Shenyang Municipal Science and Technology Bureau (1091136-9-02) |  |


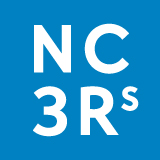


References:

1. Kilkenny C, Browne WJ, Cuthill IC, Emerson M, Altman DG (2010) Improving Bioscience Research Reporting: The ARRIVE Guidelines for Reporting Animal Research. *PLoS Biol* 8(6): e1000412. doi:10.1371/journal.pbio.1000412

2. Schulz KF, Altman DG, Moher D, the CONSORT Group (2010) CONSORT 2010 Statement: updated guidelines for reporting parallel group randomised trials. *BMJ* 340:c332.
